# Remote Microphone Hearing Aid Use Improves Classroom Listening, Without Adverse Effects on Spatial Listening and Attention Skills, in Children With Auditory Processing Disorder: A Randomised Controlled Trial

**DOI:** 10.3389/fnins.2020.00904

**Published:** 2020-08-21

**Authors:** Georgios Stavrinos, Vasiliki (Vivian) Iliadou, Menelaos Pavlou, Doris-Eva Bamiou

**Affiliations:** ^1^Ear Institute, Faculty of Brain Sciences, University College London, London, United Kingdom; ^2^Neuroscience Division, Medical School, Aristotle University of Thessaloniki, Thessaloniki, Greece; ^3^Department of Statistical Science, University College London, London, United Kingdom; ^4^Neuro-Otology Department, University College London Hospitals NHS Trust, London, United Kingdom; ^5^Biomedical Research Centre, National Institute for Health Research, London, United Kingdom

**Keywords:** auditory processing disorder, remote microphone hearing aids, spatial listening, attention, audiology, randomised controlled trial

## Abstract

**Background:**

Children with Auditory Processing Disorder (APD) often have poor auditory processing skills in the presence of normal peripheral hearing. These children have worse listening-in-noise skills compared to typically developing peers, while other commonly reported symptoms include poor attention and distractibility. One of the management strategies for children with APD is the use of Remote Microphone Hearing Aids (RMHAs), which can help improve the signal-to-noise ratio in the child’s ears. The aim of this randomised controlled trial was to examine whether RMHAs improved classroom listening in children with APD, and to further test their effects on children’s listening-in-noise and attention skills following a 6-month intervention.

**Methods:**

Twenty-six children diagnosed with APD, aged 7–12, in primary mainstream education, were randomised into the intervention (*N* = 13) and control group (*N* = 13). The primary outcome measure was the Listening Inventory for Education – Revised questionnaire, completed by children to assess their listening using RMHAs under several acoustically challenging situations in the classroom. Secondary outcome measures included the Listening in Spatialised Noise – Sentences test, assessing speech-in-noise perception and spatial listening, and the Test of Everyday Attention for Children, assessing different types of attention skills. Tests were conducted in unaided conditions. Mixed analysis of variance was used to analyse the data. The clinical trial was registered at clinicaltrials.gov (unique identifier: NCT02353091).

**Results:**

The questionnaire scores of self-reported listening skills in the classroom significantly improved in the intervention group after 3, *MD* = 7.31, *SE* = 2.113, *p* = 0.014, and after 6 months, *M* = 5.00, *SE* = 1.468, *p* = 0.016. The behavioural measures of listening-in-noise and attention did not significantly change.

**Conclusion:**

Use of RMHAs improves classroom listening, evidenced by the results of the questionnaire analysis, while a 6-month use did not have adverse effects on unaided spatial listening or attention skills.

## Introduction

Children with developmental Auditory Processing Disorder (APD) are reported to have listening difficulties in the presence of normal peripheral hearing (British Society of Audiology, 2018)^1^. Some of the most commonly reported symptoms in these children are difficulties listening in background noise and inattentiveness, also reflected in findings from behavioural tests (American Speech-Language-Hearing Association, 2005)^2^. Children with APD typically perform worse in behavioural tests measuring Speech-in-Noise (SiN) skills compared to typically developing children (Lagacé et al., 2011; Rocha-Muniz et al., 2014) and often have worse performance in sustained auditory attention compared to children suspected of APD but not meeting APD diagnostic criteria (Allen and Allan, 2014). The ability to listen in noise taps into auditory processes such as temporal ordering and binaural separation (Sharma et al., 2014), and has been found to correlate with attention (Mesgarani and Chang, 2012; Zion Golumbic et al., 2013; O’Sullivan et al., 2015) and memory (Anderson et al., 2013; Sharma et al., 2014). Spatial listening helps individuals segregate sounds arriving from different directions using auditory cues from both ears (Cameron and Dillon, 2008; Kitterick et al., 2011), a skill sometimes found to be worse in children suspected of APD compared to typically developing peers (Cameron et al., 2006; Cameron and Dillon, 2008). Adding to the difficulties, listening in the classroom is challenging as classroom acoustics can often be poor and can amplify noise and reverberation time (Shield et al., 2015).

One of the management strategies recommended to children with APD is the use of Remote Microphone Hearing Aids (RMHAs) in the classroom. The use of this system helps improve the signal-to-noise ratio for children and bypasses the negative effects of background noise and reverberation in the classroom (Johnston et al., 2009; British Society of Audiology, 2011^3^; Keith and Purdy, 2014). However, only a handful of studies examined the effects of sustained RMHA use on children with APD on specific measures (when not using the devices), such as listening in noise, language and communication, and some attention and memory skills (Johnston et al., 2009; Umat et al., 2011; Smart et al., 2018), with just one using a randomised controlled design (Sharma et al., 2012).

A study compared the scores of 10 children with APD in the Hearing in Noise test^4^ at baseline and after 5 months of daily RMHA use at school (Johnston et al., 2009). The use of RMHAs in the aided test condition at 5 months gave a gain of approximately 12 dB in the SiN test compared to unaided testing and there was a significant improvement from pre- to post-treatment on the Hearing in Noise test scores on aided testing in noise. However, this SiN improvement in the aided condition may reflect an auditory acclimatisation benefit similar to the one reported for hearing aids (Dawes et al., 2014), as there was no significant improvement between pre- and post-intervention in unaided SiN testing. In this same study there was a suggestion of improved speech perception in quiet after treatment. Even though an improvement in scores observed post-treatment in the unaided condition in quiet was not statistically significant on direct pre-post comparison, the statistically significant difference between the APD and control groups found pre-treatment was no longer discernible post-intervention, with the authors concluding that RMHA use improved the auditory system in children with APD (Johnston et al., 2009). The authors also demonstrated multiple real-life benefits, including improved hearing ability across multiple listening situations in the classroom, and improved academic status and psychosocial adjustment (Johnston et al., 2009). Another study that did not use RMHAs but low-gain hearing aids with a built-in directional microphone and noise reduction, examined changes in aided SiN skills^5^ in 14 children with APD (Kuk et al., 2008). They reported improved aided SiN test results after 6 months of hearing aid use. A more recent trial examined the ability of 28 children with APD to listen in spatial noise^6^ after using RMHAs for 5 months (Smart et al., 2018). Children were tested on two tasks, easy words and hard words, and two conditions, with and without RMHAs, at baseline and post-intervention. Results showed a significant improvement in the easy and hard words task when comparing the unaided with the aided conditions, and improved scores in the easy words task unaided post-treatment. At the same time, the scores of the Frequency Pattern Test significantly improved post-intervention, while the use of double baseline measures ruled out practice effects (Smart et al., 2018). All three trials lacked control groups in randomised design, thus further validation in controlled trials, especially in unaided conditions, is required.

Furthermore, to date only one study, cited earlier, looked into the long-term impact of RMHAs on behavioural attention tests in children with APD. Specifically, unaided sustained auditory and sustained visual attention was examined in 28 children with APD after a 5-month RMHA intervention, without showing any significant change over the intervention period (Smart et al., 2018). One other study used an attention subscale of the Children’s Auditory Performance Scale questionnaire, completed by parents at baseline and after 6 months of using hearing aids (without a remote microphone) at school (Kuk et al., 2008). While the Auditory Attention Span subscale of the questionnaire showed significantly improved scores post-intervention, it remained below what was considered normal cut-off. Parents observed their children at home, when hearing aids were not used (Kuk et al., 2008), suggesting that the improvement in the subscale scores may have been due to lasting effects on their attentive behaviours. The findings from these two studies are conflicting, but none of the trials used a randomised controlled design and a placebo or maturation effect cannot be excluded.

In the present study, we aimed to test the impact of long-term RMHA use on children with APD on self-reported listening in the classroom (assuming aided listening) and measured unaided SiN (non-spatial listening) performance. Secondary aims were to assess behaviourally whether RMHA use has any impact on unaided measured listening in spatialised noise and unaided attention skills. A questionnaire completed by children, assessing their listening through the hearing aids in different listening situations in the classroom, was used as the primary outcome measure. An uncontrolled study with children with APD using the same questionnaire (thus assuming aided listening) found improved scores following a 5-month RMHA intervention (Smart et al., 2018). Another study on a different clinical population^7^ showed that scores from the Abbreviated Profile of Hearing Aid Benefit questionnaire, also measuring listening ability through RMHAs and assuming aided listening, improved after 6 weeks of RMHA use (Rance et al., 2010). It was thus hypothesised that children with APD using RMHAs at school would give a higher total score on our study’s questionnaire compared to controls with APD. Drawing from the limited evidence of significant gain in SiN skills when tested unaided following a 5-month RMHA intervention (Johnston et al., 2009; Smart et al., 2018), it was hypothesised that a 6-month RMHA use in our study would not have a significant effect on SiN scores when tested unaided compared to scores from controls with APD.

Furthermore, there are clinical concerns that overreliance on RMHAs may bypass the need of children to pay attention to spatial cues and that this may in turn impact their spatial listening in noise performance. To examine the effect of RMHAs on spatial listening in noise in children, a spatial advantage derived measure was used, which minimises language (Cameron and Dillon, 2007) and possibly cognitive confounding factors (Tomlin et al., 2015). Despite the evidence showing that when using RMHAs listening in spatial noise is improved compared to unaided testing, there is only limited evidence of lasting benefit in unaided listening in noise after an RMHA intervention (Smart et al., 2018). In addition, the spatial listening process is still maturing in this cohort and is sound driven, as reflected in poorer spatial listening performance in children with otitis media (Tomlin and Rance, 2014). It was therefore hypothesised that use of RMHAs for a prolonged period, which bypasses the need for use of spatial perception during a substantial portion of the day, will not improve unaided spatial listening skills in children. There are also clinical concerns that prolonged use of RMHAs in the classroom may negatively impact children’s unaided attentive skills, given the passive nature of listening through RMHAs (Fey et al., 2011). To examine this, behavioural measures of auditory attention span, and divided and selective visual attention were used and tested in unaided conditions. The outcome measure of unaided sustained auditory attention was hypothesised to remain unchanged after a 6-month RMHA intervention, in view of previous findings of no effect on unaided sustained attention (Smart et al., 2018). Finally, the outcome measures of divided attention and selective visual attention have not been previously studied and were thus expected to remain unaffected by a 6-month RMHA use.

## Materials and Methods

### Participants

**FIGURE 1:**
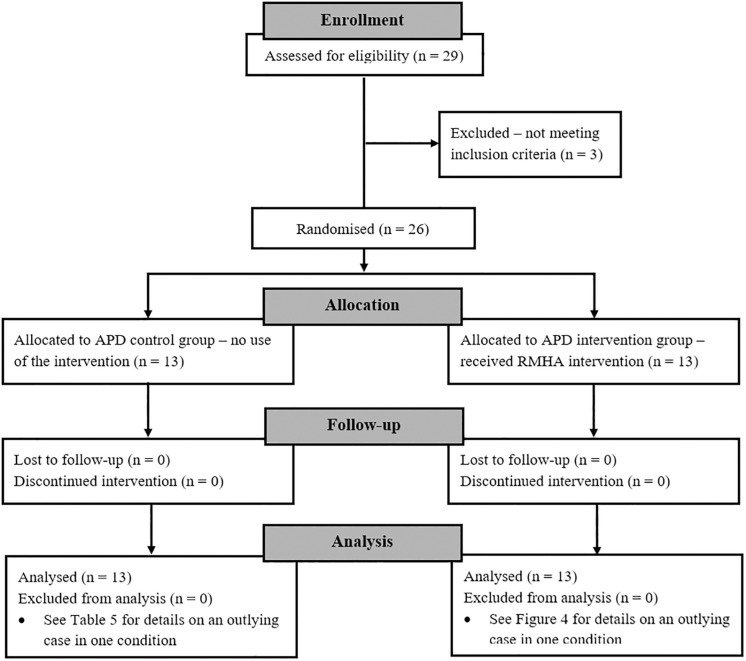
Flow diagram showing the attrition through the different stages of the clinical trial. Three children were excluded after enrolment as they did not meet the inclusion criteria (see relevant section), while there was no loss of participants due to follow-up. APD, Auditory Processing Disorder; RMHA, Remote Microphone Hearing Aid.

A sample size calculation was conducted using the F test for repeated measures between-within interaction Analysis of Variance (ANOVA) on G^∗^Power program (Faul et al., 2007), based on an estimate 0.27 effect size f^8^, 80% power, at 5% significance, using 2 groups and 3 measurement points. This calculated a total sample size of *N* = 24. To account for a possible drop-out rate (Hickey et al., 2018) of 5%, the final sample size comprised 26 children. Figure 1 outlines the attrition throughout the different stages of the trial, from enrolment to analysis, in the form of a flow diagram.

An Intention-to-Treat analysis was followed, which prevents overestimation of the effect size and helps maintain a sample size in accordance with the sample size calculation (Gupta, 2011). There were 8 girls and 18 boys, aged 7:3 (years: months) to 11:7 with a mean age of 9:8. All participants were native English speakers. Table 1 outlines the means and Standard Deviations (SDs) of age, non-verbal cognitive ability scores, and Pure Tone Audiogram (PTA) scores.

**TABLE 1:** Means and SDs of age, performance in the WNVSA, mean PTA score of all frequencies for each ear, and gender distribution for each group.

	**Gender distribution**	**Mean age in years: months (*SD*)**	**WNVSA Mean standard score (*SD*)**	**PTA-R Mean dB (*SD*)**	**PTA-L Mean dB (*SD*)**
Control group	4 females 9 males	9:8 (16.3 months)	107.0 (11.1)	6.4 (3.3)	6 (3.5)
Intervention group	4 females 9 males	9:7 (16.1 months)	99.3 (8.7)	7.3 (2.8)	7.2 (3.4)

*PTA-L, Pure Tone Audiometry – Left ear; PTA-R, Pure Tone Audiometry – Right ear; SD, Standard Deviation; WNVSA, Wechsler Non-Verbal Scale of Ability.*

#### Inclusion Criteria and Design

The inclusion criteria of the study were adopted from the APD diagnostic protocol used in Great Ormond Street Hospital in London (Great Ormond Street Hospital, 2018)^9^, which follow the APD position statement of the American Speech-Language Hearing Association (American Speech-Language-Hearing Association, 2005) and the guidelines of the European APD consensus group (Iliadou et al., 2017). The criteria aim to minimise the confounding effects of age, cognitive and hearing ability, co-occurring disorders and language on the diagnosis of APD.

The inclusion criteria used are the following:

1. Diagnosis of APD based on routine clinical tests, administered by qualified audiologists, and requiring the following conditions:
  (a) Reported parental SiN and other listening difficulties.
  (b) Normal peripheral hearing and middle ear function; i.e., air conduction PTA below 20 dB in all octave frequencies between 250 and 8 KHz (British Society of Audiology, 2012)^10^, middle-ear pressure between −150 to +50 daPa, middle-ear admittance between 0.3–1.6 cm^3^, and ear-canal volumes between 0.4 and 1.0 cm^3^ (British Society of Audiology, 2013)^11^.
  (c) Abnormal performance (at the 1st percentile score as per UK norms) on the Auditory Figure Ground (AFG) SiN subtest of the SCAN-3 C test (Keith, 2009), and
  (d) Abnormal performance (−2 SDs from the normative mean) on at least one auditory processing test: Dichotic Digits Test (DDT) (Musiek et al., 1991), Gaps-in-Noise (GiN)/random gap detection pattern tests (Keith, 2000; Musiek et al., 2005; Shinn et al., 2009), Frequency Pattern Test/duration pattern test (Musiek, 1994); or a score of −3 SDs from the normative mean on only one auditory processing test (any test from conditions “c” and “d”), and/or abnormal performance (−2 SDs from the mean) on the Spatial Advantage and Total Advantage or High-cue Speech Reception Threshold (SRT) subtests of the Listening in Spatialised Noise – Sentences (LiSN-S) test (Cameron and Dillon, 2007).
2. No developmental delay, neurological or pervasive disorder. Diagnosis of Attention Deficit Hyperactivity Disorder, Epilepsy, Autism Spectrum Disorder, Developmental Language Disorder, Down Syndrome^12^.
3. Non-verbal cognitive ability score of 85 or more^13^.
4. Aged between 7–12 years^14^.
5. Native English speakers^15^.
6. No prior use of RMHAs.

A randomised controlled trial design was used where 26 children with APD were randomised into two parallel groups, the intervention and the control group. Stratified randomisation was used (Kang et al., 2008) to balance the two groups for the strata of age (9:4 or younger, and 9:5 or older) and gender (boys, girls), as these factors might influence auditory processing performance and attentional networks (Coch et al., 2005; Yathiraj and Vanaja, 2015). The two groups were constructed using permuted blocks within each stratum, having block sizes of 3, 5, 6, and 12. Allocation ratio was 1:1 and allocation was performed using a computer generator of random numbers. This resulted in each group having 4 girls and 9 boys, with mean ages of 9:8 for the control group and 9:7 for the intervention group (see Table 1 for summary). Children in the intervention group used the RMHAs for 6 months at school, while the ones in the control group did not use any type of intervention during this period. Children in both groups were tested at baseline, at 3 months and at 6 months. Enrolment, and random allocation to groups were performed by the first author.

### Intervention

The RMHA system used in the trial was the Micro-mic coupled with the ReSound Up hearing aids, manufactured by GN ReSound. The Micro-mic broadcasts at a frequency of 2.4 GHz that wirelessly connects to the open fitting ear receivers. The two devices can stay connected within a range of 25 m.

Children in the intervention group were using binaural ear receivers, which were wirelessly connected to the microphone worn by the teacher. During the school visit, children, teachers, supporting school staff, and parents were given instructions on how to wear, connect and handle the device, along with a troubleshooting guide. Children and teachers were asked to use the RMHAs daily during school time, 5 days per week for 6 months, inside the classroom and to remove them during physical education and breaks to avoid damaging. The researcher communicated electronically with parents from both groups every month to address any issues related to use of the system, to reduce the risk of participant withdrawal, and to ensure that children in neither group were using other interventions (e.g., auditory training). There was no blinding neither for the participants nor for the researcher.

### Ethical Approval

The Bloomsbury Research Ethics Committee reviewed and approved the study. Children and their parents received information sheets and had enough time to study them at home and were encouraged to ask any questions they had. Children then signed assent forms in the presence of their parents and their parents signed consent forms, prior to the start of the study. During the researcher’s school visit, teachers were also provided with an information sheet, encouraged to ask questions and then signed consent forms.

### Tests and Procedure

Children were assessed for APD in the Great Ormond Street Hospital APD clinic prior to recruitment, following the diagnostic criteria outlined earlier. The questionnaire and behavioural outcome measures of this trial described below were administered at University College London Ear Institute on a separate date. First, children were tested on the Wechsler Non-Verbal Scale of Ability (WNVSA) (Wechsler and Naglieri, 2006), only on their first visit. They were then given the Listening Inventory for Education – Revised (LIFE-R) questionnaire (Anderson et al., 2011)^16^ to complete on each of the three visits. In addition, in these visits they were administered two behavioural tests; the LiSN-S test (Cameron and Dillon, 2007) and the Test of Everyday Attention for Children (TEACh) (Manly et al., 1999). All tests described below were administered in a quiet carpeted room without the use of the RMHAs, with only the questionnaire asking children to retrospectively assume aided listening during their RMHA use in the classroom.

#### Non-verbal Cognitive Ability Test

The WNVSA minimises the influence of possible language difficulties on the measured cognitive ability (Wechsler and Naglieri, 2006). The two subtests administered from the WNVSA did not involve any auditory stimuli (Wechsler and Naglieri, 2006), thus the total score was not expected to be influenced by auditory processing deficits. Instructions were communicated both verbally and non-verbally, while the researcher was aware of the listening difficulties exhibited by APD children in noisy situations and always ensured that instructions were understood before proceeding with the scoring. The two subtests used were Matrices (measuring general ability and perceptual reasoning) and Spatial Span (measuring general ability and visual working memory). Each subtest was calculated in *T* scores, which were then summed together to create a standard score (mean of 100, SD of 15) reflecting an overall Intelligence Quotient (IQ) score (Wechsler and Naglieri, 2006). The test duration was approximately 20 min.

#### Questionnaire

Children completed the LIFE-R questionnaire, which assesses how well the student listens to the teacher in the classroom under nine different acoustically challenging conditions (Anderson et al., 2011). While the original LIFE-R questionnaire had 15 questions, some were excluded from this study as they did not meet the aims of the study^17^, which also helped shorten the time children spent to complete the questionnaire. The nine remaining questions assessed how well children could hear the teacher when listening through traffic noise, listening when the teacher’s back was turned, when there was noise inside the classroom, noise outside the classroom, when the teacher moved around the room, when there was competing speech, when children worked in groups, when it was quiet, and during assembly (Anderson et al., 2011). The LIFE-R Total Score was calculated as the sum of all these nine conditions. The questions were specific to each group of the study, meaning that children in the control group were asked how well they could hear their teacher in these listening situations (without any mention of hearing aids), whereas children in the intervention group were asked how well they could hear their teacher in these listening situations through the RMHAs. The LIFE-R is a non-standardised questionnaire, thus the scores collected and analysed were raw scores. Children required about 10–15 min to complete this questionnaire.

#### Listening-in-Noise Test

The LiSN-S test assesses listening-in-noise and listening-in-spatialised-noise skills in children. There are four test conditions and in each up to 30 different sentences can be presented. Children listen to a target sentence among competing speech and they are asked to repeat back the sentence or as many words from that sentence as they can. In every condition, the relative position of distractors in relation to target speech changes (either 0° or 90°) in combination with the pitch (either distractor voices being same or different than the target). Test Condition 1 has different voices at ± 90°, Test Condition 2 has same voices at ± 90°, Test Condition 3 has different voices at 0°, and Test Condition 4 has same voices at 0°. Five different scores are calculated that include three derived measures that may control for language, learning and cognitive factors (Cameron and Dillon, 2007; Tomlin et al., 2015). Table 2 explains how these five scores are calculated and what they assess. In the analysis we grouped together the three LiSN-S scores that measure spatial listening in noise ability (High-cue SRT, Spatial advantage, and Total advantage) to examine the impact of the intervention on this skill. The two other scores (Low-cue SRT and Talker advantage) were grouped together during analysis as they assess non-spatial SiN skills. Testing was performed without the use of RMHAs, thus measuring unaided listening in noise skills.

**TABLE 2:** The five LiSN-S scores and what they assess (Cameron and Dillon, 2007).

**LiSN-S score**	**Calculation method**	**Description and type of assessment**
Low-cue SRT	The score of Test Condition 4.	The competing speech comes from the same direction (i.e., from the front) and has the same voices as the target speech. Assesses SiN ability when pitch and spatial cues are eliminated.
High-cue SRT	The score of Test Condition 1.	The competing speech is spatially separated and has different voices from the target speech. Assesses the ability to understand speech when both spatial cues and talker characteristics are available to help segregate the target.
Talker advantage	The difference between the scores of Test Conditions 3 and 4.	Assesses the ability of the child to use differences in talker characteristics to help segregate the target.
Spatial advantage	The difference between the scores of Test Conditions 2 and 4.	Assesses the advantage the child receives when distracting voices are moved from the front (same source as the target speech) to the side (different source from the target speech).
Total advantage	The difference between the scores of Test Conditions 1 and 4.	Assesses the same abilities as the High-cue SRT condition (i.e., ability to use spatial and pitch cues to focus on the target speech).

*LiSN-S, Listening in Spatialised Noise – Sentences; SiN, Speech-in-Noise; SRT, Speech Reception Threshold.*

During this test the child was seated opposite the tester wearing Sennheiser HD125 headphones, which were connected via the external Phonak soundcard to a ProBook HP laptop facing away from the child to minimise distractions. The special soundcard kept the competing speech constant at 55 dB Sound Pressure Level and the front target sentences at an initial 62 dB Sound Pressure Level. The front target sentences were then adjusted automatically, 2 dB up if more than 50% of the sentence words were incorrect, or 2 dB down if less than 50% of the sentence words were incorrect. If the correct sentences were exactly at 50% then there was no change in dB (Cameron and Dillon, 2008). The five scores (see Table 2) were calculated automatically in the LiSN-S software after test completion and were presented in the form of standardised dB scores and z scores.

The LiSN-S version used to test participants was the North American one. A recent study compared the performance of 48 British children, aged 6–10 tested with the North American version against the performance of the LiSN-S North American norms on 72 children of the same age group (Murphy et al., 2019). No significant differences in the two samples were found in any of the LiSN-S scores, except for the Talker Advantage score. This required a correction factor of + 0.035 in z score in the British sample (Murphy et al., 2019), a correction adopted in our study.

#### Attention Test

The four subtests used from the TEACh measure skills in Sustained Auditory Attention (Sus-AA), Divided Auditory-Visual Attention (Div-AVA), Selective Visual Attention (Sel-VA), and Divided Auditory Attention (Div-AA) (Manly et al., 1999). Table 3 outlines this information and provides a task description for each TEACh subtest. All tests were administered without the use of RMHAs.

**TABLE 3:** The four TEACh subtests, the type of attention they measure and their task description (Manly et al., 1999).

**TEACh subtest**	**Type of attention**	**Task description**
Sky search	Sel-VA	Children were required to find and circle visual targets among other visual distractors in an A3 paper.
Score	Sus-AA	Children had to count sounds with long irregular gaps in between the sounds.
Sky search dual task	Div-AVA	Children had to repeat the visual task of the Sel-VA subtest, while simultaneously count sounds like they did in the Sus-AA subtest, with the difference being that the gap in between the sounds was now fixed and shorter than previously.
Score dual task	Div-AA	Children were required to count sounds, as they did in the Sus-AA task, while simultaneously find one target word in each of the news reports that were being played.

*Div-AA, Divided Auditory-Attention; Div-AVA, Divided Auditory-Visual Attention; Sel-VA, Selective Visual Attention; Sus-AA, Sustained Auditory Attention; TEACh, Test of Everyday Attention for Children.*

During the TEACh test, the child sat opposite the tester and a ProBook HP laptop, connected to two Creative Inspire T10 speakers, was used. The speakers were facing the child from the front centre. To set the presentation level at an average of 60 dB Sound Pressure Level, a 10-second warble sound was measured from the child’s seat at ear level with a calibrated Casella CEL-450C sound level meter at an average 60 dB Sound Pressure Level. Raw scores for each test were initially collected and then converted into scaled scores (mean of 10, SD of 3) based on the normative data from the test manual (Manly et al., 1999).

The total duration of the three tests (LIFE-R, LiSN-S, TEACh) was approximately 1 h 15 min, with regular breaks given to children. Test order was randomised for each child to minimise order effects.

#### Statistical Analysis

Data were analysed in SPSS 22 statistics software, using mixed ANOVA, with group as the between-subjects factor (two levels; control and RMHA group) and time the within-subjects factor (three levels; baseline, 3 and 6 months). The assumptions of normal distribution, absence of outliers, homogeneity of variances, and equality of covariance matrices were tested. All assumptions were met for all conditions unless otherwise stated in the results.

## Results

Table 4 summarises the mean values and SDs of the control and intervention groups at baseline, at 3 and at 6 months, for all the outcome measures (i.e., the LIFE-R Total Score, the five LiSN-S scores, and the four TEACh subtests).

**TABLE 4:** The mean scores and SDs of the LIFE-R Total Score, the LiSN-S scores, and the TEACh subtests, of the two groups at all three time-points.

	**Mean (*SD*)**					
	**Control group**			**RMHA group**		
	**Baseline**	**3 months**	**6 months**	**Baseline**	**3 months**	**6 months**
LIFE-R						
Total score	30.92 (4.46)	33.33 (5.60)	30.83 (5.06)	28.31 (7.44)	35.62 (3.45)	33.31 (7.66)
LiSN-S						
Low-cue SRT	−0.56(0.91)	−0.47(1.43)	−1.38(3.34)	−0.99(1.12)	−0.63(1.62)	−1.05(1.52)
High-cue SRT	−0.84(1.46)	−0.70(1.34)	−1.23(1.83)	−1.28(1.03)	−0.60(1.09)	−0.68(1.66)
Talker Advantage	−1.02(1.15)	−0.33(1.01)	−0.44(1.12)	−0.84(0.81)	−0.60(0.83)	−0.69(0.94)
Spatial Advantage	−1.51(1.55)	−1.32(1.42)	−0.98(1.77)	−0.98(1.08)	−0.99(1.58)	−0.53(1.36)
Total Advantage	−0.57(1.34)	−0.52(1.29)	−0.63(1.07)	−0.94(1.12)	−0.48(1.06)	−0.30(1.29)
TEACh						
Sus-AA	9.77 (4.00)	7.77 (3.68)	8.23 (4.23)	6.31 (2.63)	6.62 (3.84)	5.77 (1.88)
Div-AVA	6.62 (3.18)	5.23 (2.83)	5.92 (3.25)	2.38 (2.40)	5.15 (3.67)	5.77 (2.17)
Sel-VA	9.69 (2.02)	10.31 (3.61)	10.92 (1.93)	6.00 (2.86)	8.31 (2.72)	9.62 (2.33)
Div-AA	9.15 (3.13)	8.62 (4.21)	8.69 (4.17)	6.54 (3.13)	6.77 (2.55)	6.08 (3.17)

*For the LiSN-S, disordered performance: ≤ −2; borderline performance: > −2 and ≤ −1; and normal performance: > −1. And for the TEACh, disordered performance: ≤ 4; borderline performance: > 4 and ≤ 7; and normal performance: > 7. The LIFE-R does not include cut-offs for abnormal performance as it is a non-standardised questionnaire. Div-AA, Divided Auditory Attention; Div-AVA, Divided Auditory-Visual Attention; LIFE-R, Listening Inventory for Education – Revised; LiSN-S, Listening in Spatialised Noise – Sentences; RMHA, Remote Microphone Hearing Aid; SD, Standard Deviation; Sel-VA, Selective Visual Attention; SRT, Speech Reception Threshold; Sus-AA, Sustained Auditory Attention; TEACh, Test of Everyday Attention for Children.*

### Audiometry and Non-verbal Cognitive Ability

All children had hearing thresholds below 20 dB hearing level in each frequency between 250 and 8 KHz (British Society of Audiology, 2012). From the initial enrolment of 29 children, 3 were excluded as they did not meet the inclusion criterion of scoring above 85 on the WNVSA test. The 26 children that scored above this threshold gave a mean composite full scale score of 102.5 on the WNVSA test (Wechsler and Naglieri, 2006). After group randomisation the controls had a mean score of 107.0 on the WNVSA test, while the intervention group had a mean score of 99.3 (see Table 1), with the difference being marginally non-significant, *t*(24) = 1.95, *p* = 0.063, *d* = 0.76, 95% Confidence Interval (CI) [−0.04, 1.55].

### LIFE-R Questionnaire

**FIGURE 2:**
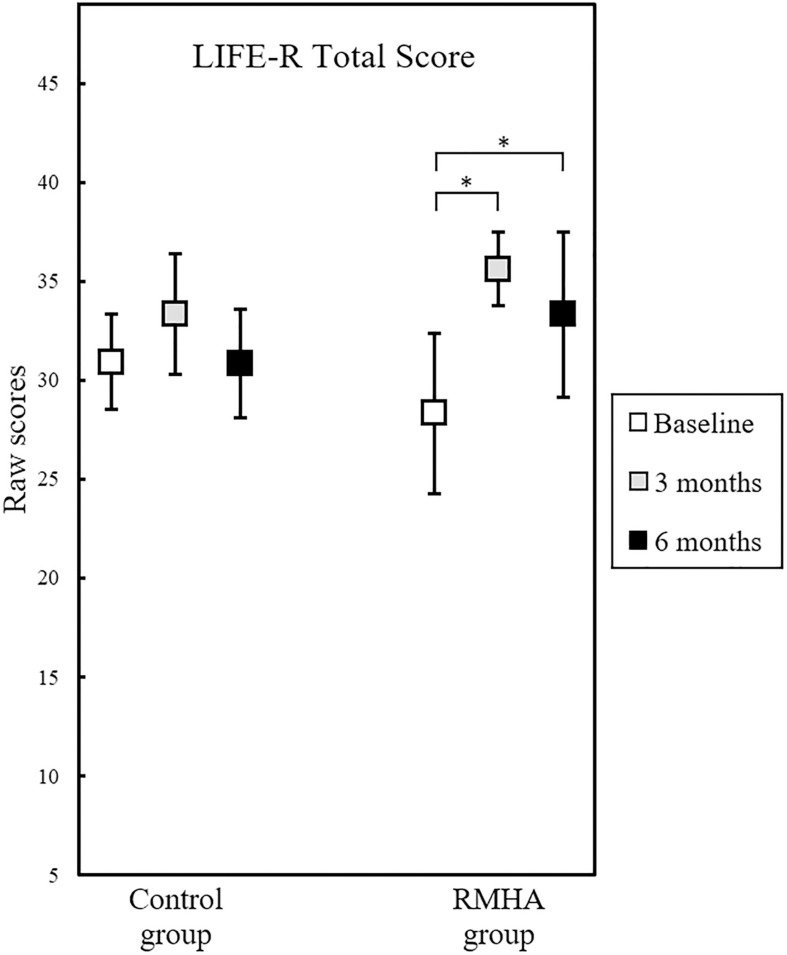
Plots of mean scores in the LIFE-R Total Score, for 12 controls and 13 RMHA children, at baseline, 3 and at 6 months. Error bars represent a 95% CI of the mean. The scores of the intervention group significantly improved from baseline to 3 months, as well as from baseline to 6 months. This was a non-standardised questionnaire, therefore there was no indication of a cut-off for abnormal performance. CI, Confidence Interval; LIFE-R, Listening Inventory for Education – Revised; RMHA, Remote Microphone Hearing Aid. ^∗^*p* < 0.05.

The LIFE-R Total Score had a significant interaction between group and time, *F*(2, 46) = 51.79, *p* = 0.042, η^2^*_p_* = 0.129, as highlighted in Figure 2.

This interaction was followed up by examining the simple main effects of group and time. There was no statistically significant difference in scores between the two groups at any of the three time points, but there was a statistically significant effect of time in the Total Score in the RMHA group only, *F*(2, 24) = 181.41, *p* = 0.002, η^2^*_p_* = 0.408. To determine the time-point of these differences pairwise comparisons were run, showing significantly improved scores in the RMHA group from baseline to 3 months, Mean Difference (*MD)* = 7.31, Standard Error (*SE*) = 2.113, *p* = 0.014, and significantly improved scores, as well, from baseline to 6 months, *MD* = 5.00, *SE* = 1.468, *p* = 0.016. Table 5 summarises these results.

**TABLE 5:** The *p*-values, partial eta squared (η^2^*_p_*) effect size, and CIs of the effect size, of the LIFE-R Total Score.

**LIFE-R**	**Interaction**		**Simple main effect**			
	Group*Time *p-*value	**η^2^*_p_* [95% CI]**	**Group *p-*value**	**η^2^*_p_* [95% CI]**	**Time *p-*value**	**η^2^*_p_* [95% CI] MD [95% CI]**
Total Score (Outlier removed)	0.042*	0.129 [0.000,0.290]	0.304 (baseline) 0.228 (3 months) 0.355 (6 month)	0.046 [0.000,0.267] 0.062 [0.000,0.292] 0.037 [0.000,0.253]	0.093^†^ (**Controls**) 0.002** (**RMHA**) 0.014* (0–3 months) 0.016* (0–6 months) 0.721 (3–6 months)	**0.217 [0.000,0.435]** **0.408 [0.081,0.588]** 7.308 [1.434, 13.182] 5.000 [0.921, 9.079] −2.308 [−7.502, 2.886]
Total Score (Outlier not removed)	0.022*	0.147 [0.000,0.387]	0.364 (baseline) 0.116 (3 months) 0.246 (6 months)	0.034 [0.000,0.243] 0.100 [0.000,0.335] 0.056 [0.000,0.278]	0.443 (**Controls**) 0.002** (**RMHA**) 0.014* (0–3 months) 0.016* (0–6 months) 0.721 (3–6 months)	**0.055 [0.000,0.234] 0.408 [0.081,0.588]** 7.308 [1.434, 13.182] 5.000 [0.921, 9.079] −2.308 [−7.502, 2.886

An outlier at the 3-month time-point (at −3.25 SDs) was removed, but an additional analysis with the outlier included was run to ensure no significant effects were generated due to outlier removal. Findings from both analyses are presented here and are identical for the simple main effect of time of the RMHA group. This is because the removed outlying case was from the control group, thus its inclusion or exclusion did not affect the outcome or interpretation of the results of the LIFE-R Total Score, showing the same significant effects in the RMHA group as in the analysis with the outlier removed. CI, Confidence Interval; LIFE-R, Listening Inventory for Education – Revised; MD, Mean Difference; RMHA, Remote Microphone Hearing Aid. ^†^p < 0.10, *p < 0.05, **p < 0.01.

### LiSN-S Test

**FIGURE 3:**
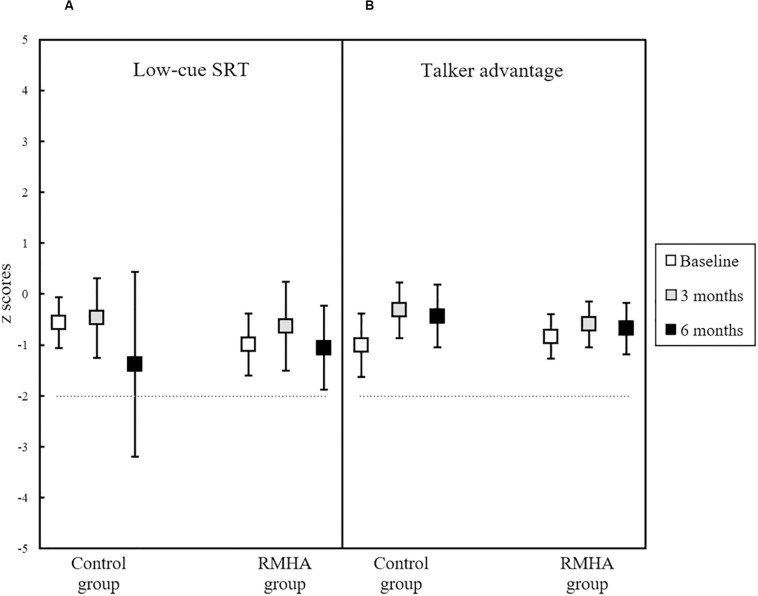
Plots of mean z scores in **(A)** the Low-cue SRT score, and **(B)** the Talker Advantage score, for 13 controls and 13 RMHA children, at baseline, 3 and 6 months. Error bars represent a 95% CI of the mean. The horizontal dotted line indicates the cut-off for what is considered abnormal performance (i.e., −2 z scores and below). CI, Confidence Interval; LiSN-S, Listening in Spatialised Noise – Sentences; RMHA, Remote Microphone Hearing Aid; SiN, Speech-in-Noise; SRT, Speech Reception Threshold.

**FIGURE 4:**
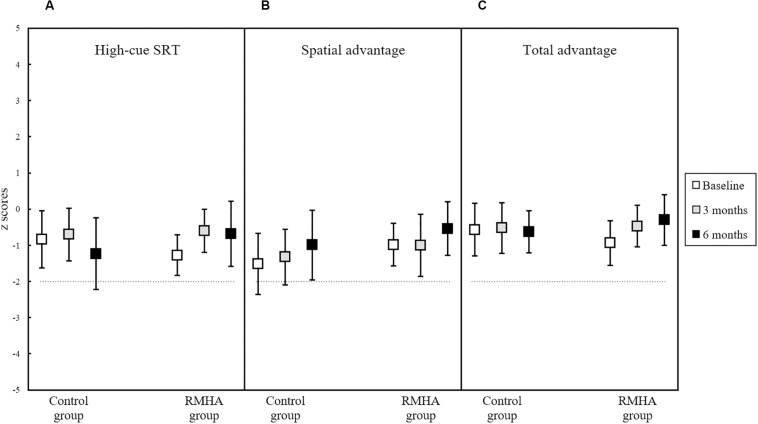
Plots of mean z scores in **(A)** the High-cue SRT score, **(B)** the Spatial Advantage score, and **(C)** the Total Advantage score, for 13 controls and 12 RMHA children (and 13 controls and 13 RMHA children in the Spatial Advantage score only), at baseline, 3 months and at 6 months. Error bars represent a 95% CI of the mean. The horizontal dotted line indicates the cut-off for what is considered abnormal performance (i.e., −2 z scores and below). One participant had two outlying cases in post-intervention testing at 6 months in the High-cue SRT and Total Advantage scores. Outliers were removed, as the participant had decreased focus and interest and had difficulty remaining seated during the fourth and final test condition. This condition is used to calculate both the High-cue SRT and Total Advantage scores, and because it is the last condition it can indicate auditory fatigue or declining attention in the subject (National Acoustic Laboratories, 2010). Removal of this case resulted in a comparison of 13 controls against 12 RMHA children in the analyses of these two conditions. CI, Confidence Interval; LiSN-S, Listening in Spatialised Noise – Sentences; RMHA, Remote Microphone Hearing Aid; SRT, Speech Reception Threshold.

None of the LiSN-S scores had a significant interaction between group and time, meaning that no treatment effect was observed. The results of the two scores assessing non-spatial SiN skills (Low-cue SRT and Talker Advantage), are summarised in Figure 3, while Figure 4 presents the results of the three LiSN-S scores measuring listening-in-spatialised-noise skills through the three scores of Spatial Advantage, High-cue SRT, and Total Advantage.

Even though the High-cue SRT and Total Advantage scores did not have a significant effect (see Table 6), a visual observation of these two scores in Figure 4 points to a small but non-significant increase in the RMHA group only over the study period. We also note a small but non-significant increase in mean z scores in the Spatial Advantage score in both groups over time.

**TABLE 6:** The *p*-values, η^2^*_p_* and CIs of the effect size, of the five LiSN-S scores.

**LiSN-S**	**Interaction**		
	Group*Time *p-*value	**η^2^*_p_***	**95% CI**
Low-cue SRT	0.562	0.019	0.000, 0.115
High-cue SRT	0.270	0.055	0.000, 0.191
Talker advantage	0.512	0.027	0.000, 0.137
Spatial advantage	0.937	0.003	0.000, 0.052
Total advantage	0.417	0.037	0.000, 0.160

*CI, Confidence Interval; LiSN-S, Listening in Spatialised Noise – Sentences; RMHA, Remote Microphone Hearing Aid; SRT, Speech Reception Threshold.*

### TEACh Test

**FIGURE 5:**
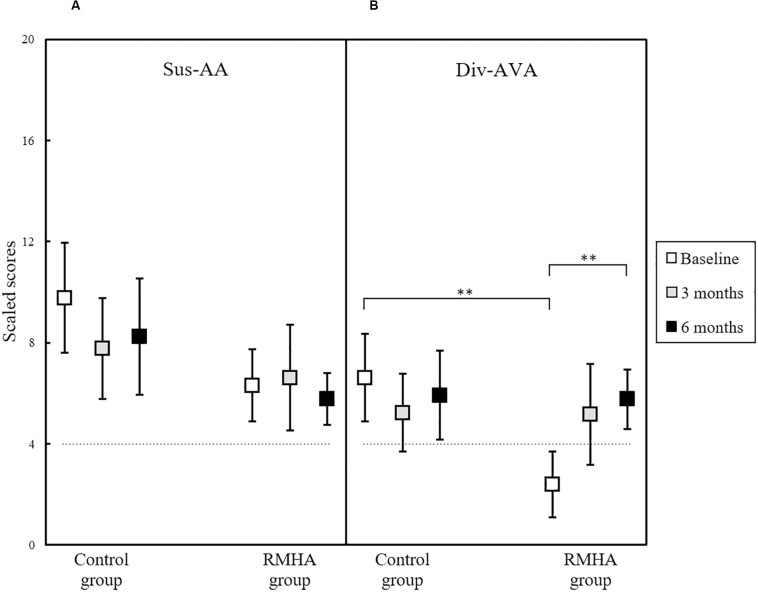
Plots of mean scaled scores in **(A)** the TEACh Sus-AA subtest, and **(B)** the TEACh Div-AVA subtest, for 13 controls and 13 RMHA children, at baseline, 3 and 6 months. Error bars represent a 95% CI of the mean. There was a significant difference between the two groups in the Div-AVA baseline scores, and significant difference in the Div-AVA scores of the intervention group from baseline to 6 months. The horizontal dotted line indicates the cut-off for what is considered abnormal performance (i.e., 4 scaled scores and below). CI, Confidence Interval; Div-AVA, Divided Auditory-Visual Attention; RMHA, Remote Microphone Hearing Aid; Sus-AA, Sustained Auditory Attention; TEACh, Test of Everyday Attention for Children. ^∗∗^*p* < 0.01.

There was a significant interaction in the Div-AVA condition, *F*(2, 48) = 4.63, *p* = 0.014, η^2^*_p_* = 0.162, as seen in Figure 5.

Performing a follow-up analysis to determine where these differences laid, we found a statistically significant difference in scores between the two groups at baseline, *F*(1, 24) = 14.68, *p* = 0.001, η^2^*_p_* = 0.380, meaning that the control group had significantly better scores at baseline than the intervention group. We also found a statistically significant effect of time in the Div-AVA scores of the intervention group, *F*(2, 24) = 5.78, *p* = 0.009, η^2^*_p_* = 0.325, but not in the scores of the control group (see Table 7 for details). To determine the time-point of this significant difference, pairwise comparisons were run, which revealed that in the RMHA group, Div-AVA scores statistically improved from baseline to 6 months, *MD* = 3.385, *SE* = 0.721, *p* = 0.002, while from baseline to 3 months no significant change was noted.

**TABLE 7:** The *p*-values, η^2^*_p_* and CIs of the effect size, of the four TEACh conditions.

**Type of attention**	**Interaction**		**Simple main effect**			
	
	Group*Time *p-*value	**η^2^*_p_* [95% CI]**	**Group *p-*value**	**η^2^*_p_* [95% CI]**	**Time *p-*value**	**η^2^*_p_* [95% CI] MD [95% CI]**
Sus-AA	0.242	0.057 [0.000, 0.192]	–	–	–	–
Div-AVA	0.014*	0.162 [0.007, 0.325]	0.001** (baseline) 0.953 (3 months) 0.888 (6 months)	0.380 [0.086, 0.583] 0.000 [0.000, 0.006] 0.001 [0.000, 0.032]	0.492 (**Control**) 0.009** (**RMHA**) 0.121 (0–3 months) 0.002** (0–6 months) 1.000 (3–6 months)	**0.057 [0.000, 0.239] 0.325 [0.027, 0.523]** 2.769 [−0.578, 6.117] 3.385 [1.381, 5.338] 0.615 [−2.678, 3.909]
Sel-VA	0.074^†^	0.103 [0.000, 0.256]	–	–	–	–
Div-AA	0.781	0.010 [0.000, 0.087]	–	–	–	–

The assumption of homogeneity of variances was violated in the Sus-AA and Div-AVA conditions. For both subtests, data transformations were not able to meet the assumption of homogeneity of variances, while other assumptions would also become violated. Violation of this assumption of homogeneity of variances increases the probability of a Type I error, but when sample sizes are equal and normally distributed a Type I error is less likely to occur (Box, 1953; Nordstokke and Zumbo, 2007). For these reasons, analysis for both subtests was carried out without adjustments. CI, Confidence Interval; Div-AA, Divided Auditory Attention; Div-AVA, Divided Auditory-Visual Attention; MD, Mean Difference; RMHA, Remote Microphone Hearing Aid; Sel-VA, Selective Visual Attention; Sus-AA, Sustained Auditory Attention; TEACh, Test of Everyday Attention for Children. ^†^p < 0.10, *p < 0.05, **p < 0.01.

**FIGURE 6:**
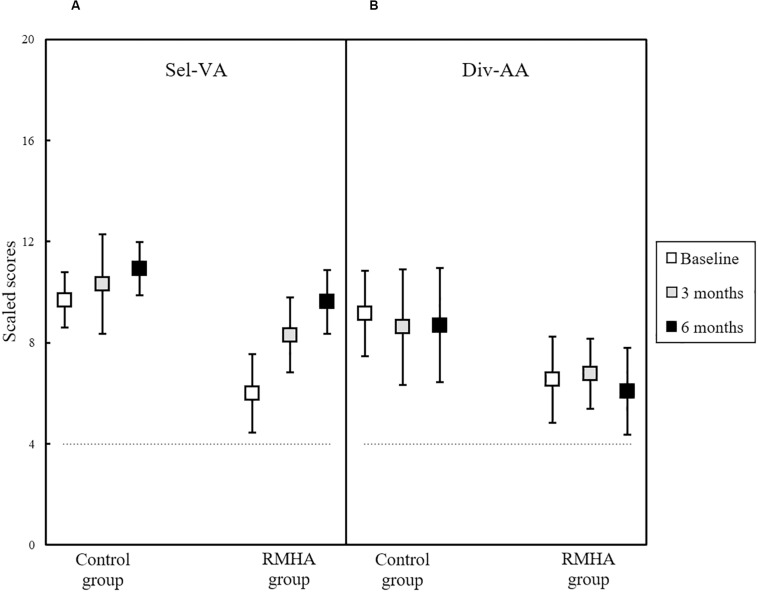
Plots of mean scaled scores in **(A)** the TEACh Sel-VA subtest, and **(B)** the TEACh Div-AA subtest, for 13 controls and 13 RMHA children, at baseline, 3 and 6 months. Error bars represent a 95% CI of the mean. The horizontal dotted line indicates the cut-off for what is considered abnormal performance (i.e., 4 scaled scores and below). CI, Confidence Interval; Div-AA, Divided Auditory Attention; RMHA, Remote Microphone Hearing Aid; Sel-VA, Selective Visual Attention; TEACh, Test of Everyday Attention for Children.

The Sus-AA subtest, seen previously in Figure 5, and the Sel-VA and Div-AA subtests shown in Figure 6 did not present with significant effects.

#### Exploratory *post hoc* Analyses

As there were significant differences in Div-AVA scores between the intervention and control group at baseline, an exploratory *post hoc* analysis controlling for baseline values was conducted. A multiple regression analysis was used including the Div-AVA baseline scores along with the group variable as independent variables, and the Div-AVA scores at 6 months as the dependent variable. After controlling for the baseline values, the intervention group had on average a (non-significant) 1.665 higher scaled score than the control group, 95% CI [−0.948, 4.279], *p* = 0.200 (see Table 8 for details).

**TABLE 8:** Unstandardised coefficients and their CIs, *t*-values, and *p*-values of each independent variable in the multiple regression model.

**Variable**	**Unstandardised coefficients**		***t***	***p*-value**	**95% CI for *B***	
	***B***	**SE**			**Lower bound**	**Upper bound**
Constant	3.078	1.406	2.189	0.039	0.170	5.987
Treatment group variable	1.665	1.263	1.318	0.200	−0.948	4.279
Div-AVA baseline scores	0.430	0.184	2.337	0.029	0.049	0.811

*CI, Confidence Interval; Div-AVA, Divided Auditory-Visual Attention; SE, Standard Error.*

To further understand the baseline imbalance, an exploratory *post hoc* examination of the auditory processing test scores used to diagnose children with APD, was conducted. Independent samples *t*-tests were run to compare the mean scores between the control and intervention group in the AFG 8 dB, AFG 0 dB, DDT average, and GiN tests. The DDT Right and DDT Left ear were averaged for each group, as there was no difference between them in any of the groups; *M* = −0.559 z score, 95% CI [−1.357, 0.239], *t*(9) = −1.584, *p* = 0.148 for the control group, and *M* = 0.444 z score, 95% CI [−2.194, 3.083], *t*(10) = 0.375, *p* = 0.715 for the intervention group. The Frequency Pattern Test was not completed by enough children in the intervention group (only two vs. eight in the control group), thus no further analysis was conducted on this test. As shown in Table 9, no significant differences were observed in these four tests between the two groups.

**TABLE 9:** Results of the independent samples *t*-tests comparing the mean differences between the control and intervention group in the AFG 8 dB, AFG 0 dB, DDT, and GiN tests.

					**95% CI of MD**	
	***N* in each group(control vs. intervention)**	***t***	***p*-value**	**MD**	**Lower**	**Upper**
AFG 8 dB	6 vs. 10	–0.427	0.676	–0.232	–1.399	0.934
AFG 0 dB	10 vs. 6	–1.013	0.328	–0.434	–1.353	0.485
DDT	10 vs. 10	1.153	0.264	0.897	–0.736	2.530
GiN	10 vs. 6	1.231	0.238	0.614	–0.456	1.685

*Differences in the sample size in each group and test occur, as some children did not complete some of the auditory processing tests during their hospital visits for reasons not disclosed in their hospital letters. AFG, Auditory Figure Ground; CI, Confidence Interval; DDT, Dichotic Digits Test; GiN, Gaps-in-Noise; MD, Mean Difference.*

## Discussion

### Classroom Listening

We found improved children-rated listening in the classroom after 3 and 6 months of RMHA use, whereas the control group did not record significant changes during this period. Previous research supports this finding, as children with APD exhibited a significant improvement from pre- to post-treatment on the Hearing in Noise test scores on aided testing in noise after a 5-month intervention period (Johnston et al., 2009). This same uncontrolled study found significant improvement in the scores of three of the ten questions from the previous version of the LIFE-R questionnaire (Johnston et al., 2009). The three questions that showed significant improvement in that study asked children how well they could hear their teacher’s voice through the hearing aids (a) when the teacher was talking in front of the room, (b) when the teacher was talking with his/her back turned, and (c) when other students were making noise inside the classroom (Johnston et al., 2009). Another uncontrolled study also found the overall LIFE-R score to improve in a group of children with APD after 5 months of RMHA use (Smart et al., 2018). The findings in both studies along with our results indicate that use of RMHAs improves perceived listening in the classroom under acoustically challenging situations. However, maturation effects due to unstandardised LIFE-R scores, a Hawthorne effect^18^, a placebo effect (Misra, 2012), or observer bias cannot be excluded.

### Listening-in-Noise Skills

The Low-cue SRT and Talker Advantage scores of the LiSN-S measuring SiN abilities did not show significant improvement over time, implying no change in the underlying ability of unaided listening in speech noise. Another likely explanation for the lack of significant improvement in these scores would be the fact that baseline performance was already within what is considered normal range for the LiSN-S test, making it difficult to observe substantial improvement post-treatment.

Unaided spatial listening skills (measured through the Spatial Advantage, High-cue SRT and Total Advantage scores) remained unchanged following a 6-month RMHA intervention, as initially hypothesised. Results from a recent study demonstrated change in unaided testing in the easy words task of an unstandardised listening-in-spatialised-noise test in children with APD over 5 months of RMHA use, while the hard words task did not change (Smart et al., 2018). Moreover, a non-randomised controlled study on stroke patients showed that a 10-week RMHA intervention significantly improved the scores of the intervention group in a sentences-in-spatial-noise test, both under unaided and aided testing, whereas the scores of the control group did not exhibit change post-intervention in either of the two conditions (Koohi et al., 2017). However, the population in that study was different from the population in the present trial (i.e., adult stroke patients with acquired APD vs. children with developmental APD), while selection bias might have affected their findings. Consistent with the findings of these two previous studies, we observed slight but non-significant increase in the High-cue SRT and Total Advantage mean z scores in the intervention group only (see Figure 4 and mean scores in Table 4). These results may be partly explained by the increased fidelity with which the brainstem responds to speech consonant-vowel stimuli, as demonstrated in a 1-year RMHA interventional study on children with dyslexia (Hornickel et al., 2012). However, our findings should be interpreted with caution because of the small effect size and wide confidence interval.

Clinical concerns that the use of RMHAs for a prolonged period may bypass the need for spatial perception because of overreliance, which may in turn impede spatial listening skills in children with APD, can be addressed by our findings of no adverse effects of treatment. This could be due to the children using the RMHAs at school only, or to the fact that they would still need to use spatial cues when attending to other voices in the class, different from the voice of the teacher. More randomised controlled research is required to evaluate spatial listening skills in RMHA intervention periods longer than 6 months.

### Attention Skills

There was no lasting effect on children’s auditory attention span, measured through the Sus-AA subtest (unaided). A previous study that used hearing aids with a built-in directional microphone intervention for 6 months on children with APD, demonstrated significant improvements in a subscale score of the Children’s Auditory Performance Scale parental questionnaire (Kuk et al., 2008). However, that study did not use remote microphones and lacked a randomised controlled design, while the improved scores reported by parents could have been the result of a placebo, Hawthorne (Snowling and Hulme, 2003; Misra, 2012), or maturation effect. It is unlikely that a passive intervention, such as hearings aids or RMHAs (Fey et al., 2011), on their own would produce lasting effects on a higher-order function, such as sustained attention. Our results agree with findings from a recent uncontrolled study that showed no change in unaided sustained auditory and sustained visual attention scores in children with APD following a 5-month RMHA intervention (Smart et al., 2018). Alternatively, the non-significant results in the Sus-AA subtest could potentially be explained by the mismatch between dynamic real-life sustained attention situations and monotonous lab-based sustained attention tasks (Head and Helton, 2015). Further RMHA intervention trials should investigate differential Sus-AA tasks with inclusion of dynamic linguistic stimuli similar to the ones that children come across in the classroom.

Similarly, the other three types of attention skills measured here (Sel-VA, Div-AA, Div-AVA) also did not present with significant change during the 6 months of RMHA use in unaided testing. This further adds to the previous finding of lack of lasting benefit due to RMHA use in an unaided sustained visual attention test (Smart et al., 2018). Even though children in the intervention group started with significantly worse Div-AVA scores at baseline in comparison to children in the control group, after 6 months these lower scores in the intervention group showed significant improvement. However, after adjusting the baseline imbalance, the differences were no longer significant. Thus, any initial observed improvements in Div-AVA scores prior to controlling for baseline can be attributed to a regression to the mean (Bland and Altman, 1994). Children in the two groups did not differ in terms of their auditory processing scores, but the difference in the WNVSA test scores between the two groups was close to significant (*p* = 0.063). While the baseline imbalance in the Div-AVA score between the two groups could be attributed to chance, performance in the WNVSA test cannot be excluded as a likely contributing factor to this difference, especially given the correlation of this Div-AVA task with a visuo-spatial processing IQ task (Manly et al., 1999). Therefore, future randomised controlled trials should consider using general non-verbal cognitive ability performance as an additional control factor during randomisation of participants into groups.

### Clinical Implications

Overall, the results of our study could provide clinicians dealing with APD useful information when drawing their management plans. The use of RMHAs, could be recommended to children with auditory processing difficulties to help improve their classroom listening, without the risk of negatively influencing their spatial listening skills, at least not after the first 6 months of RMHA use. In combination, these children could use auditory training to improve their SiN skills, based on evidence from previous research on children with APD (Cameron and Dillon, 2011; Cameron et al., 2012; Filippini et al., 2012; Loo et al., 2016). For children facing attention deficits, our findings do not reveal long-term effects from RMHA use in the form of neuroplastic changes on attention, but the effect of RMHAs during use (i.e., in aided conditions) on attention skills has not yet been studied. It may be that attention is supported while the RMHA system is in use. Other forms of management, such as auditory training, may be more suited to train children’s attention skills long-term. Recommendations from other populations facing attention problems, such as children with Attention Deficit Hyperactivity Disorder (Wolraich et al., 2011), may be adapted to fit an APD management plan. Further recommendations based on individual test deficits, have been previously reported by the authors (Stavrinos et al., 2018).

### Limitations and Future Research

While this study did not include aided test conditions (aiming to keep a relatively short test session), future research could incorporate both aided and unaided testing in behavioural attention tasks for direct comparisons between these two conditions. Other interventions such as auditory training may be better at producing neuroplastic changes in children with APD in higher-order functions, such as attention. It would be interesting to further extend previous research (Sharma et al., 2012) and directly compare different interventions against each other, such as RMHAs and auditory training, to reveal the effects each one has on children’s development. Future research could also explore the possibility of including a placebo group in RMHA trials for a short period first, to observe how it is received, before engaging participants in long-term RMHA placebo trials. Lastly, the extrapolation of research findings is restricted to groups of children who meet the same APD criteria as the ones used in this trial (see inclusion criteria in section “Materials and Methods”), which are consistent with the criteria used by the European consensus group (Iliadou et al., 2017) with the addition of the American Speech-Language-Hearing Association −3 SD criterion (American Speech-Language-Hearing Association, 2005).

## Conclusion

In a randomised controlled trial children diagnosed with APD who used RMHAs reported improved overall ability to hear their teacher through various difficult listening situations in the classroom, as attested by the questionnaire findings. Benefits were not confined only to children who scored poorly on a SiN test. Unaided spatial listening and divided attention skills were not negatively influenced by 6 months’ RMHA use. At the same time, long-term use of the intervention did not have a lasting impact on unaided sustained auditory attention skills or on any of the other three types of attention (Sel-VA, Div-AA, Div-AVA). These findings can help inform clinical APD practice by adding to the evidence of benefits of RMHA use by children with APD in the classroom (Dillon et al., 2012). Remote microphone hearing aids should still be used in combination with other environmental modifications and bottom-up management approaches (American Speech-Language-Hearing Association, 2005; British Society of Audiology, 2011), in order to maximise the quality of the acoustic input children receive during classroom time. Additional auditory training and top-down management strategies should also be considered, particularly when targeting children’s attention and memory skills. Future research can compare performance of RMHAs between aided and unaided conditions and against other interventions and should consider balancing groups in randomised controlled trials based on additional factors aside from age and gender, such as baseline non-verbal cognitive ability scores.

## Data Availability Statement

The raw data supporting the conclusions of this article will be made available by the authors, without undue reservation, to any qualified researcher.

## Ethics Statement

The studies involving human participants were reviewed and approved by the London–Bloomsbury Research Ethics Committee. Written informed consent to participate in this study was provided by the participants’ legal guardian/next of kin.

## Author Contributions

GS: conception, design and draft of the work, analysis, acquisition and interpretation of data, and revising the work critically. VI: contribution to the conception of the work, interpretation of data, and revising the work critically. MP: analysis and interpretation of the data and revising the work critically. D-EB: conception, design, and draft of the work, acquisition and interpretation of data, and revising the work critically. GS, VI, MP, and D-EB: final approval of the version to be published and agreement to be accountable for all aspects of the work in ensuring that questions related to the accuracy or integrity of any part of the work are appropriately investigated and resolved. All authors contributed to the article and approved the submitted version.

## Conflict of Interest

The authors declare that the research was conducted in the absence of any commercial or financial relationships that could be construed as a potential conflict of interest. The reviewer HD declared a past co-authorship with one of the author D-EB to the handling editor.

## Footnotes

1 http://www.thebsa.org.uk/wp-content/uploads/2017/04/APD-Position-Statement-Practice-Guidance-APD-2017.pdf

2 http://www.asha.org/policy/TR2005-00043/

3 http://www.thebsa.org.uk/docs/docsfromold/BSA_APD_Management_1Aug11_FINAL_amended17Oct11.pdf

4 Phonemically balanced sentences as a target, with speech-spectrum noise at 65 dB Sound Pressure Level matching the long-term spectrum of target sentences (Johnston et al., 2009).

5 Measured through the North Western University word-list in combination with speech-shaped noise coming from 180° that was individually adjusted (Kuk et al., 2008).

6 Words from the Lexical Neighbourhood Test were presented from the front, while a 100-talker babble recording was presented left, right, and behind the subject (Smart et al., 2018).

7 Children with Friedreich ataxia and auditory neuropathy, who had poorer auditory processing skills compared to typically developing controls (Rance et al., 2010).

8 Converted from partial eta squared effect size of 0.07, considered a medium effect (Cohen, 1988; Richardson, 2011), using the ‘Determine’ function of G^∗^Power 3 program (Faul et al., 2007). As the specific design of between-within subjects factor ANOVA with 3 measurement points and 2 groups was not previously studied using the outcome measures of this study, we assumed a medium effect size.

9 https://www.gosh.nhs.uk/medical-information/auditory-processing-disorder

10 http://www.thebsa.org.uk/wp-content/uploads/2014/04/BSA_RP_PTA_FINAL_24Sept11_MinorAmend06Feb12.pdf

11 http://www.thebsa.org.uk/wp-content/uploads/2014/04/BSA_RP_Tymp_Final_21Aug13_Final.pdf

12 Prior to APD assessment the APD clinic at Great Ormond Street Hospital requires children to undergo these assessments if there are concerns for language or attention deficits. It is possible that some children would qualify for a diagnosis of Attention Deficit Hyperactivity Disorder or Developmental Language Disorder even without the initial concerns for such deficits. This is a limitation of the set diagnostic criteria used in the hospital and hence a study limitation, too. After diagnosis of APD at the hospital, no additional assessments for these disorders were carried out for the purpose of our study due to lack of funds and time.

13 Criteria 2 and 3 aimed to minimise the possibility that the presence of APD would be influenced or caused by co-occurrence with these deficits.

14 In order to make a definitive diagnosis of APD, children need to be 7 years or older as high variability in performance and task comprehension difficulties are observed in younger children (American Speech-Language-Hearing Association, 2005; British Society of Audiology, 2011). However, the American Academy of Audiology encourages earlier assessment and intervention recommending the term “at risk” for APD in younger children (American Academy of Audiology, 2010), while the latest position statement of the British Society of Audiology stresses the need for validated APD screening tools for children younger than 6 years old (British Society of Audiology, 2018).

15 Poor language skills by non-native English speakers may influence test scores.

16 http://successforkidswithhearingloss.com/wp-content/uploads/2011/09/LIFE-R-Instruction-Manual.pdf

17 The excluded questions ask children how well they hear other students when giving answers during class, when they are on the playground/gym, when they are in the corridor, during breaks, and how well they hear the announcements from the speakers. Since the RMHA system was only used by the teacher and the participant and children were not allowed to wear it outside the classroom, these questions did not meet the study’s aim for assessing perceived aided classroom listening. The other excluded question asks children how well they hear their teacher when the overhead projector is on and making noise. This was not applicable to most classrooms as they did not have an overhead projector.

18 Over-reporting benefit due to the increased attention the treatment group receives (Snowling and Hulme, 2003).

## Abbreviations

AFG: Auditory Figure Ground
ANOVA: Analysis of Variance
APD: Auditory Processing Disorder
CI: Confidence Interval
DDT: Dichotic Digits Test
Div-AA: Divided Auditory Attention
Div-AVA: Divided Auditory-Visual Attention
GiN: Gaps-in-Noise
IQ: Intelligence Quotient
LIFE-R: Listening Inventory for Education – Revised
LiSN-S: Listening in Spatialised Noise Sentences
MD: Mean Difference
PTA: Pure Tone Audiogram
RMHA: Remote Microphone Hearing Aid
SD: Standard Deviation
SE: Standard Error
Sel-VA: Selective Visual Attention
SiN: Speech-in-Noise
SRT: Speech Reception Threshold
Sus-AA: Sustained Auditory Attention
TEACh: Test of Everyday Attention for Children
WNVSA: Wechsler Non-Verbal Scale of Ability.
